# Left atrial anterolateral linear ablation for biatrial tachycardia via Bachmann's bundle, interatrial septum, and left atrial anterior wall under mitral isthmus block

**DOI:** 10.1002/joa3.12850

**Published:** 2023-04-24

**Authors:** Takayuki Sekihara, Takafumi Oka, Kentaro Ozu, Yasushi Sakata

**Affiliations:** ^1^ Department of Cardiology, Faculty of Medical Sciences University of Osaka Osaka Japan

**Keywords:** Bachmann's bundle, biatrial tachycardia, ultra‐high‐resolution mapping

## Abstract

Biatrial tachycardia via Bachmann's bundle, interatrial septum, and left atrial anterior wall can be treated by left atrial anterolateral linear ablation without left atrial appendage isolation, even under mitral isthmus block.

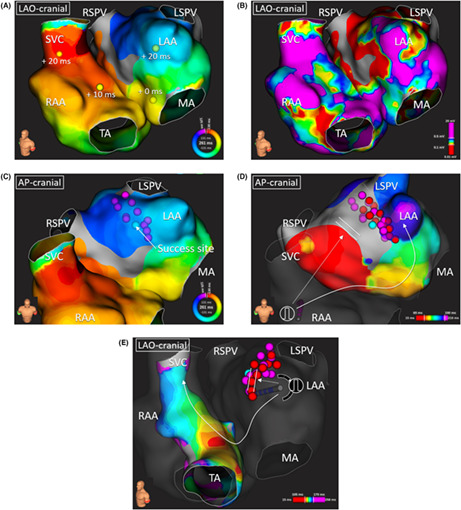

A 64‐year‐old man with a history of three prior atrial fibrillation ablations in other institutions underwent the fourth ablation because of left ventricular dysfunction caused by persistent atrial tachycardia (AT). Previous lesion sets included circumferential pulmonary vein isolation, left atrial (LA) roof line, cavotricuspid isthmus block line, lateral mitral isthmus block line, continuous fractionated atrial electrogram ablation, and nonpulmonary vein foci ablation. We initially performed ultra‐high‐resolution mapping of the AT and entrainment mapping from both the left and right atria. The AT was counter‐clockwise biatrial tachycardia (BiAT) via the Bachmann's bundle (BB), interatrial septum, and LA anterior wall (Figure 1A; Video S1). The voltage map during the AT showed low voltage areas (LVA) on the LA anteroseptal wall, likely created by previous ablations (Figure 1B). To avoid the risk of isolating the left atrial appendage (LAA), we selected the LA anterior linear ablation between the LA anterior LVA and the LA roof while sparing the area between the LVA and the mitral annulus. The BiAT was terminated during the radiofrequency (RF) applications at the LA anterior wall (30–40 W, 30–60 s for each point) targeting 20 Ω local impedance (LI) drop using IntellaNav StablePoint™ (Boston Scientific) (Figure 1C). However, the LA activation map during right atrial appendage (RAA) pacing still showed residual interatrial conduction, probably via the BB (Figure 1D **)**. We added further ablation at the BB breakthrough site and ended the session. The durability of the previous ablation lesions was confirmed during the session. We omitted the final AT inducibility check because of the exceeding overall procedure time.

**FIGURE 1:**
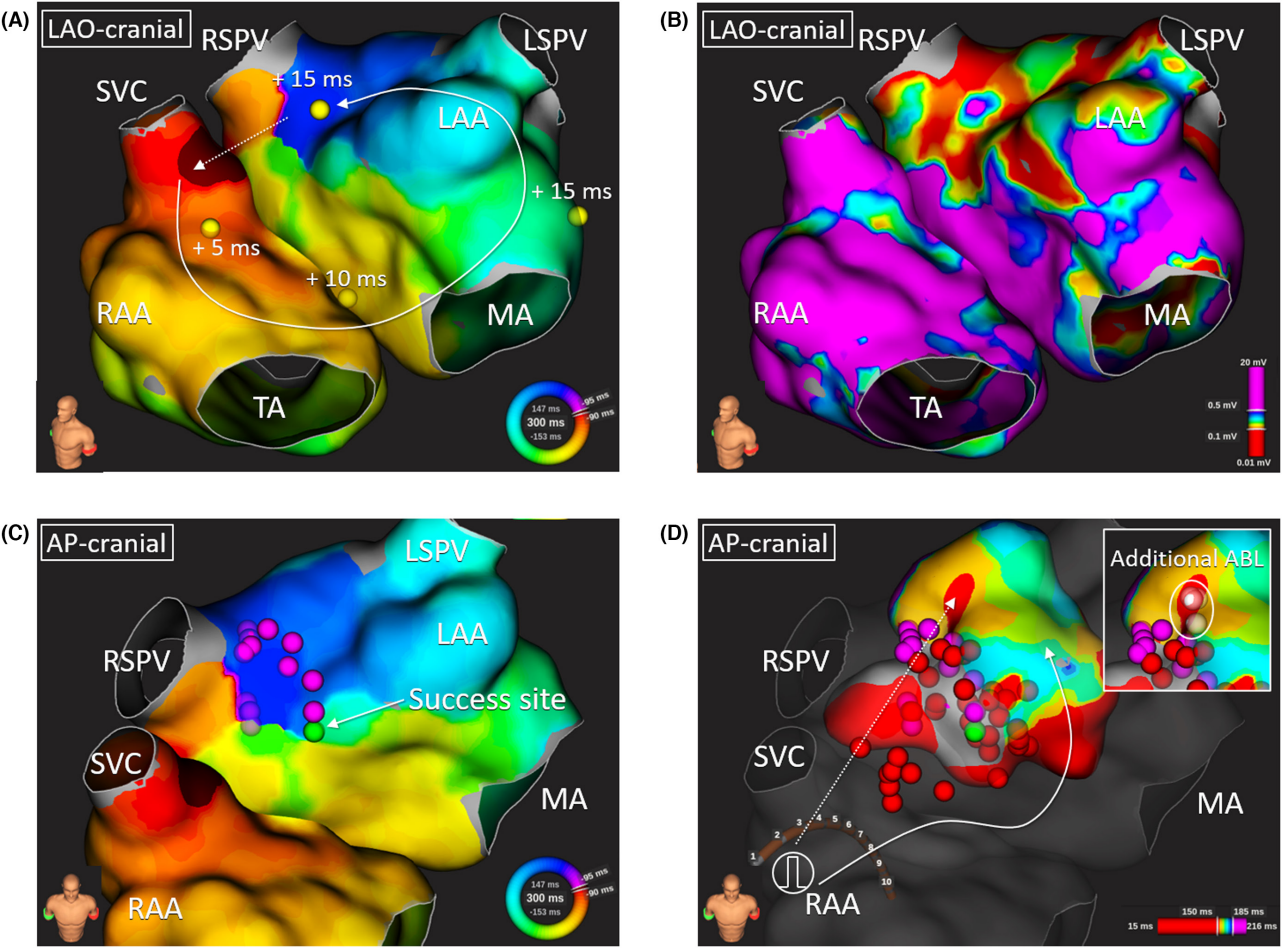
(A, B) The activation map and voltage map of the BiAT. Each value represents the postpacing interval minus tachycardia cycle length. (C) Ablation points until the BiAT termination is superimposed on the BiAT activation map. (D) The activation map during right atrial appendage pacing after the ablation presented residual Bachmann's bundle conduction (dotted arrow). Total left atrial anterior wall ablation points are superimposed on the map, and the additional ablation points after the mapping (white tags) are presented in the box. The scar threshold (Confidence mask) was set at 0.02 mV. BiAT, biatrial tachycardia; LAA, left atrial appendage; LAO, left anterior oblique; LSPV, left superior pulmonary vein; MA, mitral annulus; RAA, right atrial appendage; RSPV, right superior pulmonary vein; SVC, superior vena cava; TA, tricuspid annulus.

The AT recurred a few weeks after the fourth session, and we performed the fifth ablation. An activation map demonstrated the recurrence of BiAT (Figure 2A). The LA anteroseptal LVA and scar area became broader than that in the fourth session because of the radiofrequency deliveries described above (Figure 2B). We created a new linear lesion lateral to the previous one to distance the new lesion from the BB breakthrough site.^1^ To attain transmural lesions, we increased RF power and duration during the fifth session (max 45 W, up to 180 s at each point if the LI drop was insufficient, especially at the LVA). The recurrent BiAT was terminated during the LA anterolateral liner ablation near the LAA (Figure 2C), and we added adequate additional ablations. The LA activation map during RAA pacing and the RA activation map during LAA pacing after the ablation presented the bidirectional BB conduction block (Figures 2D,E). We also confirmed AT noninducibility with left and right atrial burst pacing up to 300/min at the end of the session. The patient's left ventricular function was normalized 2 months after the fifth session without AT recurrence.

**FIGURE 2:**
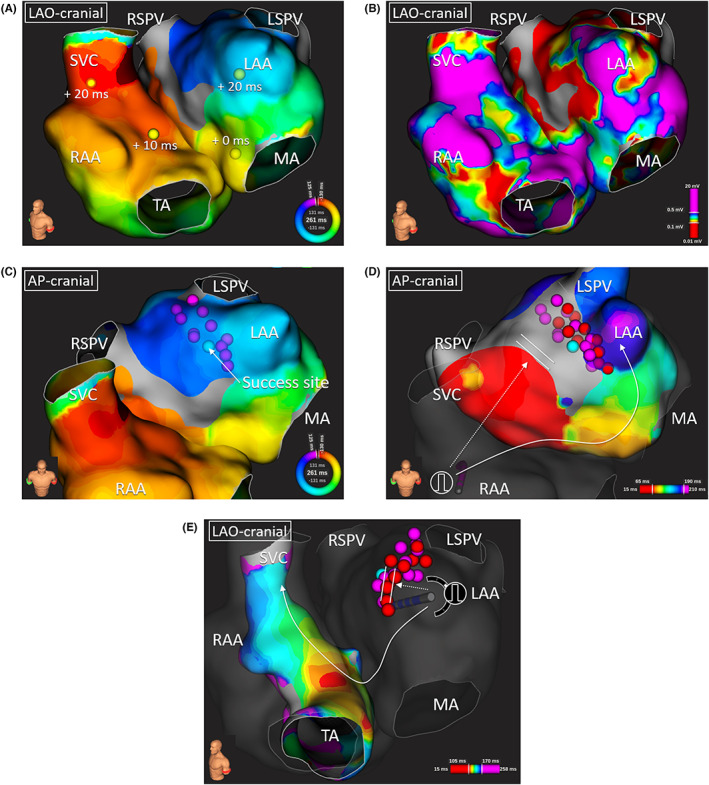
(A, B) The activation map and voltage map of the recurrent BiAT. Each value represents the postpacing interval minus tachycardia cycle length. (C) Ablation points until the BiAT termination is superimposed on the BiAT activation map. (D) The left atrial activation map during RAA pacing. (E) The right atrial activation map during LAA pacing after the ablation. SVC isolation superior to the right atrial earliest site of the map (A) was performed before acquiring map (E). Total left atrial anterior wall ablation points are superimposed on (D, E). The scar threshold (Confidence mask) was set at 0.02 mV. BiAT, biatrial tachycardia; LAA, left atrial appendage; LAO, left anterior oblique; LSPV, left superior pulmonary vein; MA, mitral annulus; RAA, right atrial appendage; RSPV, right superior pulmonary vein; SVC, superior vena cava; TA, tricuspid annulus.

This case highlights the importance of the ablation design of BiAT via Bachmann's bundle, interatrial septum, and LA anterior wall, which occurred after mitral isthmus block line ablation. The potential ablation targets, in this case, were (1) the right atrial earliest site, (2) the “superior half” LA anterior linear ablation: LA anterior LVA‐LA roof line, (3) the “inferior half” LA anterior linear ablation: LA anterior LVA‐mitral annulus line. In this case, the right atrial earliest site could have been isolated by superior vena cava isolation. However, it has been reported that BB connects to the right atrium with a broad area,^2^ and the ablation targeting the apparent breakthrough site on the activation map might fail to eliminate the connection.^3^ We chose the superior half LA anterior linear ablation to eliminate the BB conduction at the LA and avoid the LAA isolation. The inferior half LA anterior linear ablation was a possible alternative strategy. However, this area presented a higher amplitude than the superior side and presumably had a healthier conduction property than the superior side.^4^ We decided not to ablate this area to preserve the conduction toward the LAA during sinus rhythm.

Where to cut the BB conduction was also an issue; the lesion of the fourth session failed to eliminate the BiAT. In the fifth session, a new linear lesion on the lateral side of the previous one successfully blocked the residual BB conduction. We hypothesize that the new lesion was lateral to the BB conduction breakthrough site and thus facilitated the complete conduction block.^1^ On the BiAT map of the fourth session, the SKYLINE™ presented an interval of 20 ms during which both atria were silent, which may have represented the epicardial BB conduction (Figure 3). The previous LA anterior linear ablation created during the fourth session was probably located beneath the epicardial potion of the BB and failed to achieve its block (Figure 4).

**FIGURE 3:**
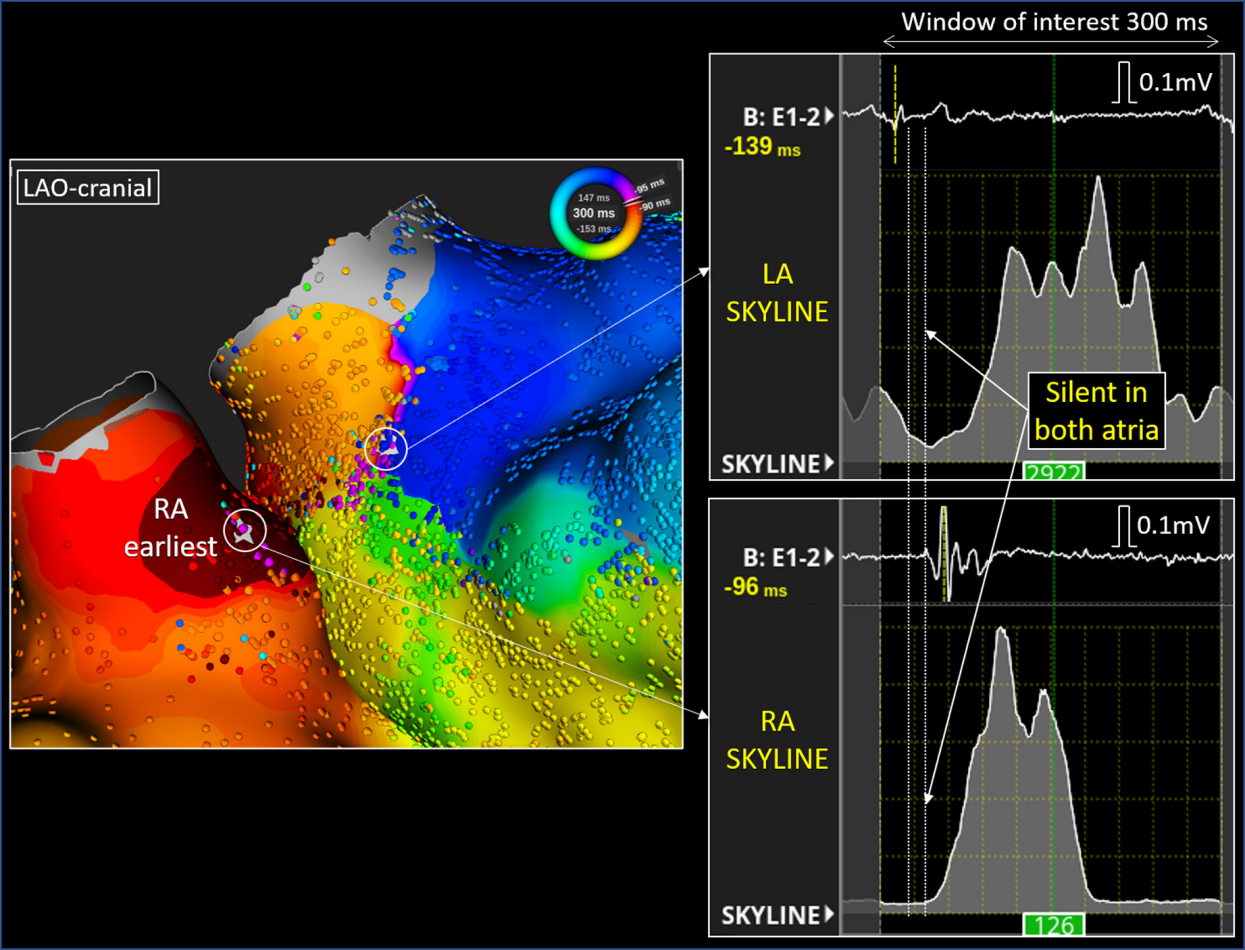
Local electrocardiograms at the right atrial earliest activation site and SKYLINE™ of the BiAT, biatrial tachycardia during the fourth session. LA, left atrium; LAO, left anterior oblique; RA, right atrium.

**FIGURE 4:**
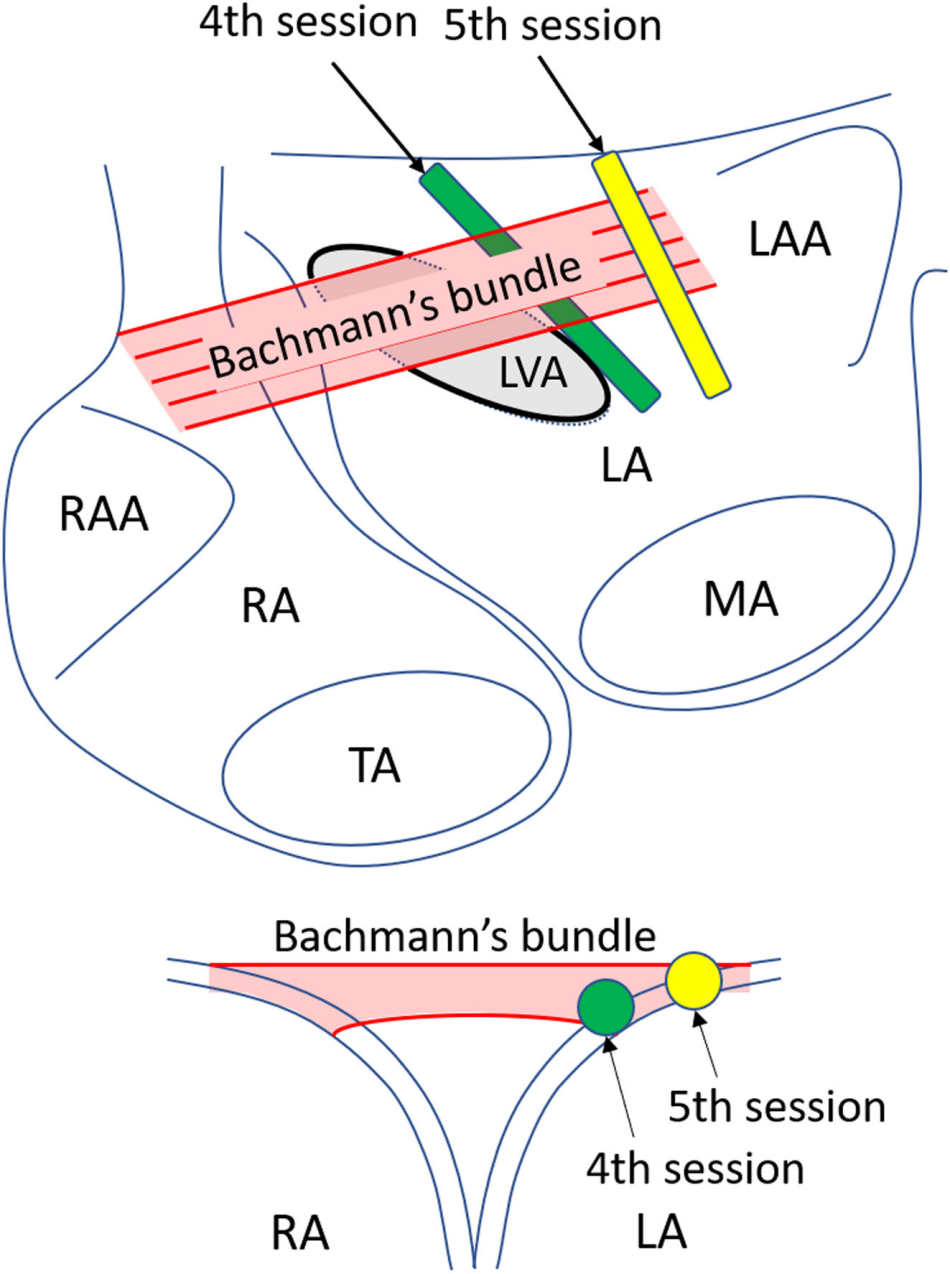
A schema of the relationship between the ablation lesions and Bachmann's bundle. LA, left atrium; LAA, left atrial appendage; LVA, low voltage area; MA, mitral annulus; RA, right atrium; RAA, right atrial appendage; TA, tricuspid annulus.

In conclusion, this case presents an ablation strategy of BiAT via BB, interatrial septum, and LA anterior wall that emerged after the mitral isthmus block. In this situation, preserving the conduction toward the LAA should be considered. Careful evaluation of the ultra‐high‐resolution map and understanding of the interatrial conduction anatomy are essential for the successful management of this type of BiAT.

## CONFLICT OF INTEREST STATEMENT

Authors declare no conflict of interests for this article.

## ETHICS APPROVAL

N/A.

## DECLARATIONS

*Approval of the research protocol*: N/A. *Informed consent*: N/A. *Registry and the Registration No*.: N/A. *Animal studies*: N/A.

## Supporting information


Video S1.

## Data Availability

Available upon reasonable request.
